# Fab Antibody Fragments to Dog Leukocyte Antigen DR (DLA-DR) Directly Suppress Canine Lymphoma Cell Line Growth In Vitro and in Murine Xenotransplant Model

**DOI:** 10.3390/cancers18010048

**Published:** 2025-12-23

**Authors:** Aleksandra Studzińska, Marek Pieczka, Angelika Kruszyńska, Leszek Moniakowski, Anna Urbaniak, Andrzej Rapak, Arkadiusz Miazek

**Affiliations:** 1 Department of Biochemistry and Molecular Biology, Wroclaw University of Environmental and Life Sciences, Norwida 31, 50-375 Wroclaw, Poland; aleksandra.studzinska@upwr.edu.pl (A.S.); anna.urbaniak@upwr.edu.pl (A.U.); 2 Hirszfeld Institute of Immunology and Experimental Therapy, Polish Academy of Sciences, Weigla 12, 53-114 Wroclaw, Poland

**Keywords:** canine lymphoma, DLA-DR, MHC II, Fab fragments, immunotherapy

## Abstract

Background/Objectives: Canine Diffuse Large B-cell Lymphoma (cDLBCL) is characterized by a high prevalence of MHC II DR (DLA-DR) antigen overexpression. Murine anti-pan-DLA-DR monoclonal antibodies (mAbs) B5 and E11 have been previously observed to promote death of cDLBCL cells in vitro and in vivo. Consequently, DLA-DR antigens are considered a prospective target for passive immunotherapy aside from CD20. While infusion of anti-pan MHC II mAbs has demonstrated tumor suppression in cDLBCL xenografted immunodeficient mice, the relative contributions of direct cellular versus immune-mediated mechanisms to this therapeutic effect remain undefined. This study aimed to dissect these potential mechanisms of mAb E11. Methods: Canine lymphoma and leukemia cell lines CLBL1 and CLB70 were incubated with full E11 antibody or its F(ab′)2 and Fab fragments and cell viability was assessed with sub-G1 assay then, NOD-SCID mice were xenotransplanted with 1.5 × 10^7^ canine CLBL1 cells expressing nanoluciferase and were infused either with mAb E11 or its fragments, each at 1 mg/kg body mass, twice weekly for three consecutive weeks. Tumor burden was monitored by assessing body weight, nanoluciferase activity in blood, and by flow cytometric analyses of bone marrow tumor cell content. Time to tumor progression (TTP) was calculated based on weight loss and luminescence measurements. Results: We observed cytotoxic activity of monovalent E11-Fab fragments in vitro and in vivo. The mean TTP for mice treated with irrelevant mouse IgG antibodies was 9.8 ± 4.65 days. In contrast, treatment with E11 Fab fragments resulted in a TTP of 19.1 ± 2.67 days, which was similar to that achieved with the full E11 mAb (19.5 ± 1.73 days) and E11 F(ab′)2 fragments (18.1 ± 2.9 days). Conclusions: Our findings demonstrate a potent antibody cytotoxicity mechanism that operates in vivo and is independent of cell surface MHC II crosslinking or Fc engagement. These data support the promising potential of E11-Fab fragments for further clinical development as a therapeutic agent in canine lymphoma.

## Simple Summary

Antibodies targeting pan-MHC class II (MHC II) epitopes have demonstrated efficacy in suppressing the growth of B-cell lymphoma in immunodeficient mouse models. However, the precise delineation between direct cellular mechanisms and immune-mediated effects responsible for this therapeutic efficacy has often remained poorly defined. Here, we present evidence that monovalent Fab fragments targeting dog leukocyte antigen DR (DLA-DR) elicit direct tumor-suppressing effects towards canine lymphoma cells. Remarkably, the magnitude of this suppression is comparable to that achieved by divalent F(ab′)2 fragments and full monoclonal antibody (mAb) counterparts. Therefore, our data reveal an antibody cytotoxicity mechanism that operates independently of cell surface MHC II crosslinking or Fc engagement and is intrinsically potent. Given their inherent advantages, including reduced immunogenicity and enhanced tissue penetrability, Fab fragments derived from therapeutic anti-pan MHC II monoclonal antibodies (mAbs) represent a compelling option for further clinical development.

## 1. Introduction

Canine lymphomas (CLs) are among the most commonly diagnosed neoplasms in the canine population. Their occurrence is mainly observed in animals between 6 and 9 years old, regardless of gender [1]. Although certain breeds exhibit genetic predisposition to the development of these malignancies [2], lymphomas can arise in dogs of any breed. The estimated incidence of CLs ranges from approximately 20 to 100 cases per 10,000 animals [3]

CLs are categorized into three main groups according to the size of neoplastic cells: small-cell lymphomas (with a slow, indolent course), intermediate-cell lymphomas (of moderate aggressiveness), and large-cell lymphomas (characterized by a rapid and aggressive progression) [4] An essential component of classification is also immunophenotypic analysis [5] and the stage of disease progression, incorporating both clinical and histological staging, which facilitates precise prognostication and treatment optimization [6]. Canine CLs display a phenotypic distribution analogous to that observed in humans, with the majority (63.8%) representing B-cell lymphomas, and smaller subsets comprising T-cell lymphomas (35.4%) or non-B/non-T-cell lymphomas (0.8%) [7]. The growing demand for more effective therapeutic approaches for CLs underscores their potential as a valuable translational research model [8,9]. Namely, the studies on CLs contribute to a deeper understanding of oncogenesis and the development of innovative therapies in humans [10].

In veterinary medicine, CLs are treated with multidrug chemotherapy protocols that are adaptations of therapies employed in human medicine. One of the most commonly used regimens is the CHOP protocol, which includes: cyclophosphamide, doxorubicin (hydroxydaunorubicin), vincristine (Oncovin), and prednisone/prednisolone [11]. The MOPP chemotherapy protocol, comprising mechlorethamine, vincristine, procarbazine, and prednisone, has been utilized in cases resistant to initial treatments [12]. In the management of CLs, CHOP and MOPP chemotherapy can be implemented with or without the incorporation of L-asparaginase [13]

Rituximab, a monoclonal antibody directed against CD20, a surface antigen expressed on most B-cell malignancies, was introduced to the pharmaceutical market in the late 1990s [14]. Rituximab is a chimeric monoclonal antibody comprising murine-derived variable regions specific for CD20 and a human IgG1 Fc domain that mediates immune effector functions. Its therapeutic activity is primarily mediated through the depletion of CD20-positive B cells via antibody-dependent cellular cytotoxicity (ADCC) and complement-dependent cytotoxicity (CDC) [15]. Long-term clinical studies and observations have confirmed that combination therapy integrating chemotherapy with targeted treatment (R-CHOP) significantly improves treatment outcomes compared to standard chemotherapy alone (CHOP). Data indicate that treatment with R-CHOP results in enhanced clinical efficacy, reflected by a higher overall response rate (85.1% vs. 72.3%), elevated complete remission rate (29.5% vs. 15.6%), and extended progression-free survival (33.1 vs. 20.2 months). Moreover, R-CHOP is associated with a superior 3-year overall survival rate (82.5% vs. 71.9%) [16].

Following the successes of rituximab therapy, attempts were made to adapt this treatment for canine lymphoma, to integrate it into veterinary clinical practice [12]. However, owing to interspecies differences in CD20 amino acid sequences and the resulting non-conservation of the rituximab target site in the canine protein, the direct use of mAbs against human CD20 in dogs has proven impractical [17]. To address this limitation, caninized anti-CD20 antibodies were developed for the treatment of B-cell lymphomas in dogs. In vitro and in vivo investigations utilizing a xenograft mouse model of the canine lymphoma cell line CLBL1 demonstrated the efficacy of the anti-canine CD20 monoclonal antibodies 1E4-cIgGB and 1E4-cIgGC. Moreover, administration of these antibodies in healthy Beagle dogs resulted in pronounced depletion of peripheral B cells [12,15,18].

In the context of alternative therapeutic targets, MHC class II (MHC-II) has emerged as a promising option for the treatment of lymphomas. Compared to CD20, MHC-II-targeted therapy has demonstrated significant potential, particularly in B-cell lymphomas, where across both species MHC-II DR is expressed on the majority of lymphoid neoplasms derived from precursor cells, with expression levels exceeding those of CD20 [19].

Constitutively expressed on antigen-presenting cells, such as monocytes, macrophages, dendritic cells, and B lymphocytes, classical MHC-II molecules play a central role in mediating the presentation of peptides from exogenous proteins to T lymphocytes. MHC-II molecules are critical for presenting antigens to CD4^+^ T lymphocytes [20] whose importance in antitumor immunity has become increasingly appreciated. Diminished MHC-II expression has been linked to adverse clinical outcomes in B-cell lymphoma, correlating with reduced overall survival [19].

In human hematologic malignancies, three murine antibodies targeting human leukocyte antigen DR (HLA-DR)—Lym-1, h1D10, and L243 (IMMU-114)—have demonstrated variable therapeutic efficacy. IMMU-114 has been administered to dogs with lymphoma without eliciting renal impairment or systemic immunodeficiency, contrary to initial concerns based on DLA-DR expression profiles. In vitro and in vivo studies conducted using the B5 mAb (anti DLA-DR) demonstrated therapeutic efficacy using immunodeficient NOD-SCID mice xenotransplanted with the canine CLBL1 cell line (a canine model of diffuse large B-cell lymphoma (DLBCL)) [21,22]. While these results confirm MHC-II as a validated therapeutic target for canine lymphoma, the mechanism of action remains insufficiently defined. Therefore, the fundamental ambiguity regarding the necessity of the Fc domain and antigen crosslinking for therapeutic success presents a critical translational gap, justifying the current study’s need to mechanistically dissect the intrinsic cytotoxic potential of monovalent DLA-DR targeting Fab fragments.

Specifically, this delineation is crucial because, according to the prevailing scientific dogma, efficient MHC II-mediated cell death is generally accepted to require crosslinking of the antigen by a bivalent antibody. Consistent with this, studies have shown that while bivalent F(ab′)_2_ fragments can induce cytotoxicity, monovalent Fab fragments typically fail to trigger detectable cytotoxic effects [23]. This prerequisite for bivalent binding creates a significant research gap regarding the true intrinsic signaling capacity of anti-MHC II antibodies. The development of next-generation antibody fragments is a critical area of recent oncology research driven by the need for agents with improved tumor kinetics. Smaller fragments, such as Fab fragments, offer inherent advantages over full-length antibodies, including reduced immunogenicity and superior tissue penetrability [24]. The overarching goal of this study was to definitively dissect the requirements for anti-DLA-DR therapeutic activity by comparing the full E11 monoclonal antibody (mAb) with its bivalent F(ab′)_2_ and monovalent Fab fragments. By using the monovalent Fab fragment—which lacks both the Fc domain (eliminating immune effector functions) and the capacity for crosslinking—we could test the cell’s capacity to undergo direct cytotoxicity through a single-site, non-aggregating interaction.

We hypothesize that a unique conformational epitope recognized by the E11 antibody can initiate cell death signaling in vivo via a Fab fragment, operating independently of both Fc-mediated effects and antigen crosslinking.

## 2. Materials and Methods

### 2.1. Enzymatic Fragmentation of Antibodies

Monoclonal antibody at 1 mg/mL was subjected to enzymatic fragmentation using immobilized papain resin (Immobilized Papain Agarose Resin, Thermo Fisher Scientific, Waltham, MA, USA) in the presence or absence of 1 mM cysteine for generating Fab or F(ab′)2 fragments, respectively. The enzymatic reactions were carried out for 5 h at 37 °C. Then, Protein A affinity chromatography was used to trap the Fc antibody fragments as well as undigested mAbs. SDS-PAGE was used to estimate the purity of the fragment preparation.

**Figure 1:**
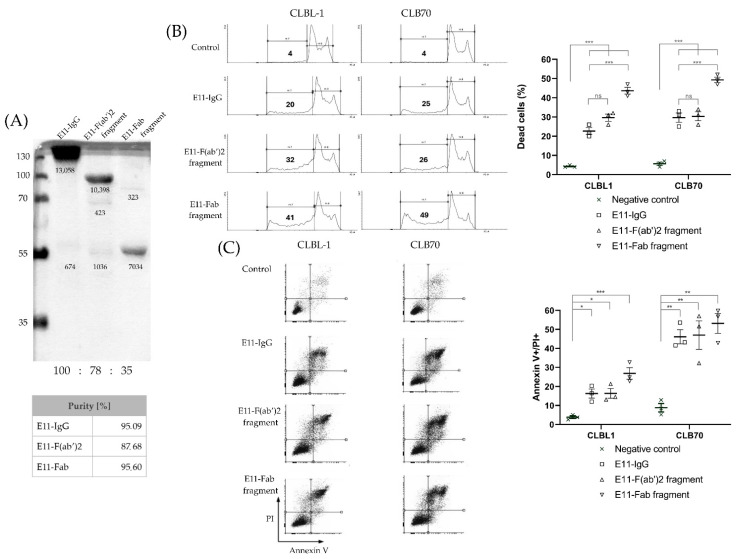
Evaluation of E11 mAb purity and cytotoxic effects after fragmentation. (**A**) SDS-PAGE confirms the molecular weights of the full E11 monoclonal antibody (mAb) and its derived fragments. The area of each band [px^2^] was measured and placed below the band. The purity details are provided. (**B**) Sub-G1 flow cytometry quantifies cell death induced by the full E11 mAb and its fragments in two canine lymphoid cell lines. The numbers indicate the percentage of cells within the electronically gated sub-G1 population. (**C**) Annexin V/PI flow cytometry assay. Numbers shown on the graphs represent the percentage of late apoptotic/necrotic double-positive cells in each replicate. Data in (**B**,**C**) are presented as mean ± SEM. Statistical significance: * *p* < 0.05, ** *p* < 0.01, *** *p* < 0.001, ns = not significant. The uncropped SDS-PAGE is shown in Supplementary Materials (Supplementary Figure S1).

Purity of the E11 antibody and its fragments was determined by densitometric analysis of the Western blot image (Figure 1A) using ImageJ 1.53 k (Wayne Rasband and contributors, National Institutes of Health, Bethesda, MD, USA). The calculation was based on the formula: Purity (%) = (Signal Area of Expected Molecular Weight Band/Total Signal Area of All Bands) × 100%.

### 2.2. Downmodulation of DLA-DR upon Binding of E11 mAb to CLBL1 and CLB70 Cell Lines

Two canine cell lines were used in this study: CLBL1 canine lymphoma cell line (at passage #2) was provided by Dr Barbara Ruetgen from the Institute of Immunology, Department of Pathobiology, University of Veterinary Medicine, Vienna, Austria [25] and the CLB70 canine leukemia cell line (at passage #3) was available in-house [26]. The cells were incubated with 10 μg/mL of full E11 mAb or its Fab fragment for 4 h at 37 °C. Then, after washing the cells with ice-cold FACS buffer (Phosphate-Buffered Saline supplemented with 2% Fetal Bovine Serum and 0.05% NaN_3_), the cells were stained with FITC-labeled B5 mAb for 20 min on ice and analyzed on BD FACS Calibur flow cytometer (Becton Dickinson, Franklin Lakes, NJ, USA). For each cell line, two biological replicates were obtained, and each included two technical replicates. Statistical analysis was performed using GraphPad Prism version 8.0.2 (GraphPad Software, San Diego, CA, USA). For each cell line differences were assessed using an unpaired, two-tailed Mann–Whitney U test.

### 2.3. In Vitro Cytotoxicity Assessment of E11-Fab and E11-F(ab′)2 Towards CLBL1 and CLB70 Cell Lines

The cells were incubated with antibodies and their fragments at concentration 10 ug/mL for 48 h, then for sub-G1 assay cells were fixed in 75% ethanol at 4 °C 30 min, and then incubated with 50 ng/mL PI staining solution and 0.2 mg/mL RNase in the dark overnight at 4 °C. For Annexin V/PI apoptosis assay cells were collected, washed twice with ice-cold FACS buffer (Phosphate-Buffered Saline supplemented with 2% Fetal Bovine Serum and 0.05% NaN_3_) and then stained with 50 ng/mL PI and 0.5 µg/mL Annexin V-Pacific Blue solution for 15 min at RT in the dark. Data was acquired on a BD LSRFortessa™ flow cytometer (Becton, Dickinson and Company, Franklin Lakes, NJ, USA) and analyzed with CellQuest™ 3.1f software (BD Biosciences, San Jose, CA, USA). Each experiment was conducted in 3 biological replicates. Statistical analysis was performed using GraphPad Prism 8.0.2 (GraphPad Software, San Diego, CA, USA) software. One-way ANOVA followed by Tukey’s post hoc test was performed to compare all groups.

### 2.4. Development of CLBL1 Cell Line Stably Transduced with a Secreted Nanoluciferase (CLBL1-sLuc)

The CLBL1 cells were cultured in RPMI1640 medium (Gibco, Thermo Fisher Scientific, Waltham, MA, USA) supplemented with 20% Fetal Bovine Serum (FBS; Gibco, Thermo Fisher Scientific, Waltham, MA, USA), 100 U/mL penicillin, and 100 μg/mL streptomycin (Biowest, Nuaillé, France) at 37 °C in a humidified atmosphere containing 5% CO2.

Transduction of the CLBL1 cells to confer the ability to produce secreted luciferase (CLBL1-sLuc) was performed using a lentiviral system (GNT-LVP377-PBS, Holzel-biotech, Köln, Germany). Lentiviral particles were added to the cells (1 × 10^6^) at a ratio of 10 μL of virus per 0.5 mL of cells. After 72 h post-transduction, antibiotic selection was initiated by changing the culture medium to complete medium containing puromycin (Puromycin dihydrochloride 10 mg/mL, Thermofisher Scientific, Waltham, MA, USA) at a concentration of 1 µg/mL.

Selection of transduced cells was carried out based on their resistance to puromycin for a period of 7 days, as well as by verifying the expression of the introduced gene, specifically by measuring the secreted luciferase production capacity. Luminescence of the cell culture supernatant was measured using the Pierce Cypridina Luciferase Glow Assay Kit (Thermo Fisher Scientific, Waltham, MA, USA). Luminescence measurements were conducted starting 72 h post-transfection, and subsequent measurements were performed every 48 h throughout the entire establishment period of the CLBL1-Luc line (32 days). A total of 20 µL of culture medium was transferred to each well of a black 96-well plate, followed by the addition of 50 µL of working solution to each well. The readings were taken 10 min after the addition of reagents, once the luminescent signal had stabilized. The readout was performed using a microplate reader (Spark; Tecan, Männedorf, Switzerland).

### 2.5. Establishment of CLBL1-sLuc In Vivo Growth in NOD-SCID Immunodeficient Mice

The Local Ethical Committee approved all animal experiments presented in this study (Approval no. 04/2023P1). NOD/SCID [27] mice were purchased from Janvier Labs (Le Genest-Saint-Isle, France) and housed in a standard SPF laboratory environment with a 12-h light/dark cycle, providing ad libitum access to food and water. The experiment was conducted on 34 mice, divided into groups of 8 animals (except for a group of 2 sham-treated naive mice). At the time of the experiment, the mice were 8 weeks old.

At the time of cell collection, the cell culture reached 80% confluence. The culture medium was collected and centrifuged at 300× *g* for 5 min to pellet the cells. The cells were then resuspended in PBS, and the centrifugation and washing were repeated three times. Finally, the cells were resuspended in PBS at a concentration of 7.5 × 10^7^ cells/mL. The average cell viability, measured using trypan blue, was 92–94%. A 27-gauge needle and a 1 mL syringe were used for cell injection. The cells were administered intravenously through the caudal vein (vena caudalis) as a single injection on day “0” of the experiment. To achieve proper visualization of the vein, the animals were placed in a cage with a heating lamp positioned above them. Subsequently, the mice were transferred to a restraining device that allows easy access to the tail vein. Each mouse received 1.5 × 10^7^ CLBL1-sLuc cells in 200 μL of PBS.

### 2.6. Antibody Infusion to Mice

On the fourth day after transplanting CLBL1-sLuc cells, the NOD-SCID mice were stratified into a control group, which did not receive any therapeutic antibodies, and experimental groups treated with either full E11 antibody, E11-Fab fragments, or E11-F(ab′)2 fragments. Randomization was performed based on individual body weight, ensuring that comparable average body weights were achieved within each group.

The first administration of the antibodies occurred on day four following the injection of tumor cells. Throughout the experiment, the antibodies were administered seven times, twice per week. The specific days of administration are indicated in the experimental timeline (blue arrows). The antibody preparations were at a concentration of 1 mg/1 mL. Prior to each administration, the mice were weighed, and the dose for each individual was calculated based on their body weight. The dosing regimen was 1 mg per 1 kg of body weight (1 μg/g body weight). After weighing the mice [g], the appropriate volume of the antibody preparation [μL] was measured and the dose was adjusted with sterile saline for injection to a final volume of 100 μL.

The control group received a similarly prepared suspension, but containing mouse irrelevant antibodies of the same isotype as E11 mAb (IgG2a) that were not directed against antigens expressed on the administered tumor cells.

The preparations were administered intraperitoneally using a 27-gauge needle and a 1 mL syringe. After proper restraint of the mice to facilitate access to the abdominal cavity, the drugs were administered in a volume of 100 μL to the lower left quadrant of the abdomen, slightly off the midline.

### 2.7. Monitoring Tumor Progression in Xenotransplanted NOD-SCID Mice

Due to the ability of the cells to produce secreted luciferase [28] it became possible to monitor tumor progression by measuring luciferase released from tumor cells into blood. Additionally, during the experiment, body weight changes in the mice were monitored, and regular clinical observations of the animals’ health status were conducted. After the experiment, bone marrow isolation was performed, and the luminescence levels were measured in the marrow suspensions as well.

The experiment was designed to last for three weeks following the implantation of tumor cells. In a subset of control-irrelevant IgG-treated mice, approximately 16 days post-procedure, a marked deterioration in clinical condition was observed, prompting the early euthanasia of these animals humanely. Euthanasia was performed by placing the mice in an anesthetic chamber, where a flow of 5% isoflurane was administered for a minimum of 5 min. Once full sedation was achieved, the mice were euthanized by decapitation [29]. During necropsy, a thorough examination of the internal organs was conducted, including an evaluation of their gross morphology and any pathological changes. Additionally, bone marrow was carefully extracted for subsequent analysis.

### 2.8. Luminescence Measurements

For the measurement of luminescence variability in blood, samples were collected from the tail vein of the mice. The animals were placed under a heating lamp, and once a stress response was observed, characterized by the animal distancing itself from the heat source, they were transferred to the restrainer. Blood collection was performed through a single puncture of the tail vein using a 30 G needle. Due to the invasiveness of blood collection, biological replicates (multiple blood draws on the same day) were not performed. For the measurement of secreted luciferase activity produced by tumor cells, analysis can be performed in all biological fluids as well as in homogenates obtained post-mortem from tissues. The results for a given sample of collected biological fluids were obtained through luminescence measurements, conducted according to the following protocol: to 10 µL of the sample, 50 µL of reagent Cypridina Luciferase Glow Assay Working Solution (Thermo Fisher Scientific, Waltham, MA, USA) was added. Luminescence readings were recorded using a luminometer within 10 min after mixing the two components. Measurements were performed using the GloMax^®^ 20/20 luminometer (Promega, Madison, WI, USA). The obtained values are expressed as relative light units [RLU].

### 2.9. Bone Marrow Isolation and Analysis

Murine bone marrow was isolated by mechanical disruption of the femurs. The femurs were separated from the hip joint, detached from the tibia, and the surrounding muscle tissue was excised. Bones were disrupted in 3 mL sterile phosphate-buffered saline (PBS) to release bone marrow cells. Following thorough homogenization, the suspension was passed through a 70 µm cell strainer mounted on a 50 mL Falcon tube, with additional PBS added to maximize cell recovery. At this stage, cells were resuspended in equal volumes of PBS, and a fraction was collected for direct luminescence analysis in bone marrow preparations. The remaining cell suspension was centrifuged at 350× *g* for 5 min at 4 °C. The pellet was resuspended in 1.5 mL Ammonium–Chloride–Potassium lysis buffer (CTS™ ACK Lysing Buffer, Thermo Fisher Scientific, Waltham, MA, USA) and incubated for 3 min at room temperature to lyse residual red blood cells. Lysis was quenched by adding PBS to a final volume of 15 mL, followed by centrifugation under the same conditions. The final cell pellet was resuspended in a FACS buffer (PBS supplemented with 2% fetal bovine serum and 0.05% sodium azide) for flow cytometry. Cell staining for analysis by flow cytometry was performed using primary anti-dog MHC class II antibodies and secondary Phytoerythrin (PE)-conjugated goat anti-mouse IgG antibodies. The control group was stained with irrelevant mouse IgG2a and secondary antibodies as above. The analysis was conducted using BD FACSLyric™ flow cytometer (Becton, Dickinson and Company, Franklin Lakes, NJ, USA). The primary B5 antibody [21] was applied to the cell suspension in the form of hybridoma cell culture supernatant in a volume of 200 µL. Incubation was performed on ice for 30 min, followed by two washes with FACS solution. The cell pellets were then resuspended in 200 µL of FACS solution, and a secondary antibody (F(ab′)_2_ Goat Anti-Mouse IgG (H + L), polyclonal, Invitrogen, Thermo Fisher Scientific, Waltham, MA, USA; lot 1923631) was added at a dilution of 1:200. The incubation was carried out on ice, in the dark, for 30 min. After incubation, the cells were washed twice with FACS solution. Until analysis, cells were suspended in 500 µL of 4% formaldehyde solution.

## 3. Results

To evaluate the purity and biological activity of E11 antibody fragments, we first conducted sodium dodecyl sulfate–polyacrylamide gel electrophoresis (SDS-PAGE). Analysis of preparations designated for in vitro and in vivo studies (Figure 1A) confirmed that the antibody preparations migrated at their expected apparent molecular weights. Only trace amounts of contaminating degradation products were detected, indicating a high level of purity. To assess the biological activity of the E11 antibody fragments, their cytotoxicity was compared to that of the full E11 monoclonal antibody (mAb) using sub-G1 and Annexin V/PI assays. These assays measured cell death in canine lymphoid tumor cell lines CLBL1 and CLB70 (Figure 1B,C). At a concentration of 10 μg/mL, the E11-Fab, E11-F(ab)_2_ fragments, and the full E11 mAb induced cell death in over 40%, 30%, and 20% of the cells, respectively, within 48 h. These findings demonstrate that the fragmentation of the E11 mAb effectively preserves its cytotoxic activity in vitro. The observed increase in cytotoxicity for the fragments compared to the full mAb is likely due to the higher molar amounts of antigen-binding sites present in the fragmented preparations at the same mass concentration.

**Figure 2:**
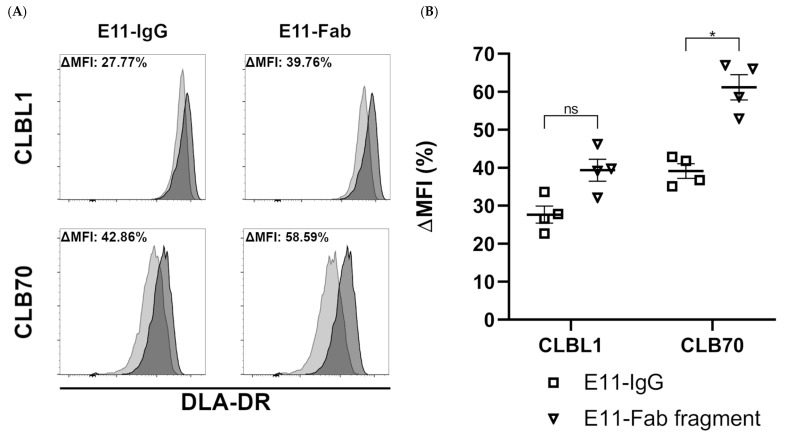
Dog leukocyte antigen DR (DLA-DR) down-regulation upon bivalent or monovalent binding of E11 mAb fragments to CLBL1 or CLB70 cells. (**A**) Percent of change in mean fluorescence intensity (MFI) relates to a difference in DLA-DR expression levels in cells untreated (black histograms) or treated (grey histograms) with the indicated antibody fragments. (**B**) Statistical analysis of DLA-DR downmodulation induced by full E11 mAb or its Fab fragment. Data are presented as mean ± SEM. Statistical significance: * *p* < 0.05, ns = not significant.

One prerequisite for MHC II-induced cell death is that antibodies must be able to downregulate MHC II DR expression upon binding their cognate epitope, as demonstrated by Vidović et al. (1995) [23]. As shown in Figure 2, both bivalent and monovalent engagement of DLA-DR by the E11 antibodies resulted in a marked downmodulation of DLA-DR cell surface expression levels after four hours of incubation. The monovalent E11 binding, however, consistently induced slightly higher DLA-DR downregulation.

**Figure 3:**
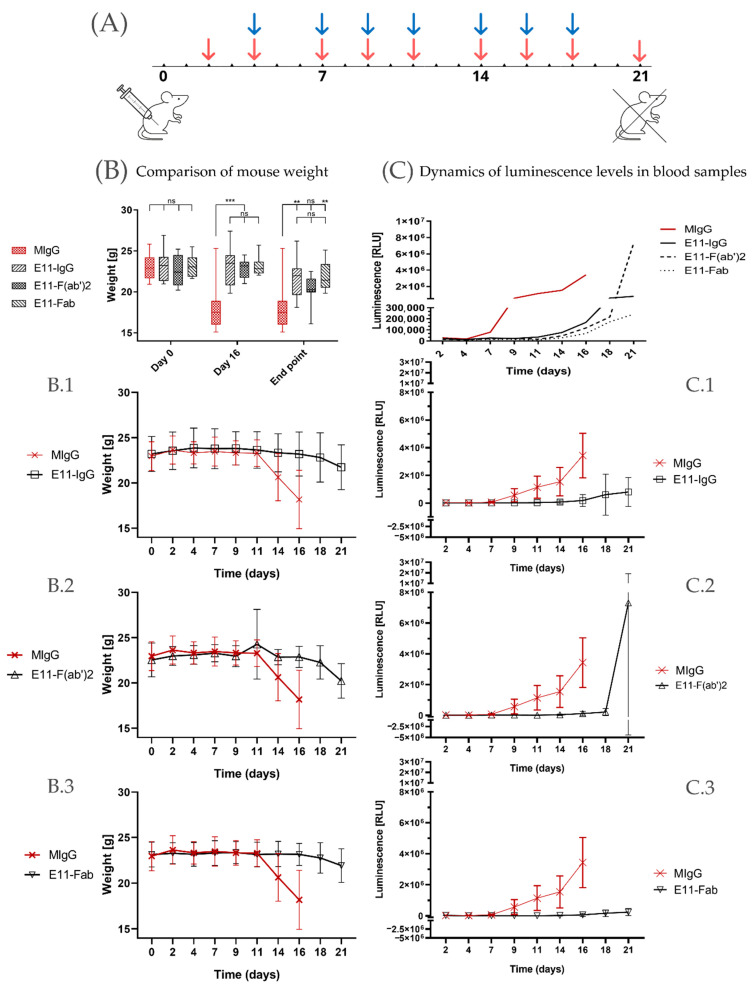
(**A**) Experimental timeline: The x-axis shows the experiment days. On day 0, mice were inoculated with CLBL1-sLuc cells. Red arrows indicate measurement days (body weight and luminescence from blood samples). Blue arrows mark antibody administration days. Day 21 indicates planned euthanasia. (**B**) Comparison of mouse weight. The graph illustrates differences in body weight of mice at selected time points during the experiment: on day 0, day 16, and at the termination of animal participation. The end point (euthanasia day) weight refers to the body mass of mice from the MIgG group that were humanely euthanized on day 16 of the experiment; the remaining animals were euthanized according to schedule on day 21 (*n* = 20) or on day 18 (*n* = 4) if their clinical condition necessitated earlier termination. Group sizes: MIgG *n* = 8, E11-IgG *n* = 8 E11-F(ab′)_2_ *n* = 8, E11-Fab *n* = 8. Statistical analyses were performed using two-way ANOVA. Data are presented as mean ± SEM. Statistical significance: ** *p* < 0.01, *** *p* < 0.001, ns = not significant. (**B.1**–**B.3**) depict the changes in body weight over time. The presented values represent the mean ± SEM for each experimental group ((**B.1**): E11-IgG, (**B.2**): E11-F(ab′)_2_, (**B.3**): E11-Fab) compared with the control MIgG group (highlighted in red). (**C**) Dynamics of luminescence levels in blood samples. The plot presents the mean luminescence values obtained for all experimental groups throughout the entire measurement period. (**C.1**–**C.3**) depict the dynamics of changes in mean values ± SEM for the experimental groups ((**C.1**): E11-IgG, (**C.2**): E11-F(ab′)_2_, (**C.3**): E11-Fab), shown in comparison with the MIgG control group (indicated in red). Group sizes: MIgG *n* = 8, E11-IgG *n* = 8 E11-F(ab′)_2_ *n* = 8, E11-Fab *n* = 8. Data are presented as relative luminescence units [RLU]. Graphical representations and statistical analyses were performed using GraphPad Prism 8.0.2.

To assess the in vivo efficacy in killing tumor cells, we infused E11-mAb and its fragments into NOD-SCID mice previously implanted with the CLBL1-sLuc, a canine B cell lymphoma cell line genetically modified to secrete nanoluciferase. The experiment was designed to span three weeks following the implantation of tumor cells (Figure 3A). During this period, disease progression was monitored through regular body weight measurements and blood luminescence readings.

To standardize the results, weight change readings were compared at three selected time points: day 0 (baseline), day 16 (at the time of experiment conclusion for the control groups), and day 21 (termination of the experiment for all mice) (Figure 3B). At the outset of the experiment, mice were assigned to groups to minimize differences in average initial body weights.

In the control group, which received irrelevant, isotype-matched mouse immunoglobulin (MIgG), a significant deterioration in clinical condition was observed approximately 16 days post CLBL1-sLuc transplantation. Notable symptoms, including substantial weight loss (approximately 15–20%), prompted the early euthanasia of these animals. Experimental groups, euthanized according to the planned schedule, 5 days after the control group, also exhibited significant weight loss by the end of the experiment. Mice treated with full E11 mAb experienced an average weight loss of 7.01%, while mice in the E11-Fab group showed a reduction of 4.78%. The greatest weight loss was observed in the E11-F(ab′)2 group, with a decrease of 12.02%.

Monitoring luminescence levels in peripheral blood over time (Figure 3C) allows for the minimally invasive assessment of lymphoma progression dynamics in vivo as the level of luciferase activity directly correlates with tumor cell burden. The initial luminescence measurements taken on day 2 post CLBL1-sLuc implantation showed a homogeneous background luciferase activity in all transplanted animals with no statistically significant differences between the groups (Supplementary Table S1). Based on the luminescence level changes over time, as shown in Figure 3C, we observed that in the irrelevant MIgG-treated group, the rise in the luminescence was observed as early as on day seven post CLBL1-sLuc implantation whereas in E11 treated groups, similar increase in the luminescence appeared on average around day fifteen and peaked within next 4–5 days (Figure 3C).

**Figure 4:**
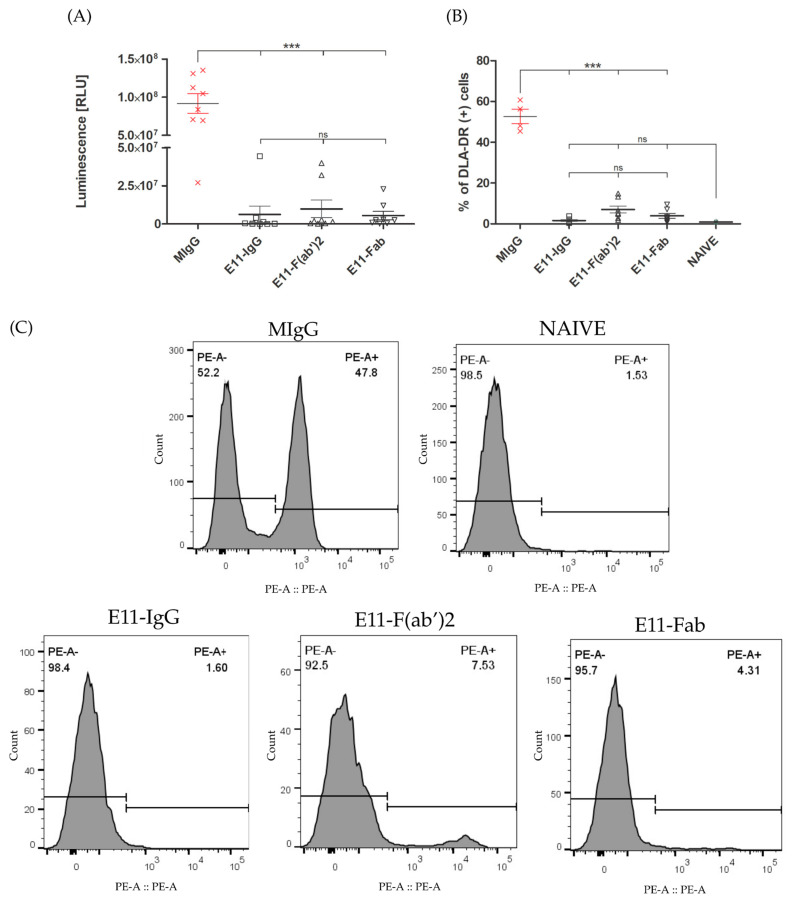
(**A**) Post-mortem luminescence measurements of bone marrow. Luminescence was measured in bone marrow homogenates and is expressed in relative light units (RLU). Group sizes: MIgG *n* = 8, E11-IgG *n* = 8 E11-F(ab′)_2_ *n* = 8, E11-Fab *n* = 8. (**B**) Quantification of DLA-DR-positive cells in the bone marrow cell fraction. The DLA-DR antigen is expressed on the surface of CLBL1 and CLBL1-sLuc cells, but is absent from murine cells. Naive (non-inoculated) mice show a background-level DLA-DR signal in flow cytometry analysis. Group sizes: MIgG *n* = 4, E11-IgG *n* = 8, E11-F(ab′)_2_ *n* = 8, E11-Fab *n* = 8, Naive *n* = 2. (**C**) Representative histograms depicting flow cytometry analysis of bone marrow cell isolates. Detection of engrafted CLBL1 lymphoma cells was based on the expression of canine major histocompatibility complex class II (MHC II; DLA-DR), using the primary monoclonal antibody B5 (anti-DLA-DR), followed by a PE-conjugated secondary antibody (goat anti-mouse IgG). The PE-A^+^ population corresponds to DLA-DR-positive (CLBL1: DLA-DR^+^) cells. Data are presented as mean ± SEM. Individual data points represent results from individual mice. Statistical significance: *** *p* < 0.001, ns = not significant. Graphical representations and statistical analyses (two-way ANOVA) were performed using GraphPad Prism 8.0.2 (GraphPad Software, San Diego, CA, USA), while flow cytometry data were processed and analyzed with FlowJo v10 software.

The osteotropic potential of the CLBL1-Luc cell line was previously established by its consistent formation of metastatic bone foci in NOD-SCID mice [21]. We assessed the therapeutic efficacy of E11 antibody preparations by quantifying tumor spread in the bone marrow of treated mice, using both luciferase activity and CLBL1-sLuc cell counts. The control group, treated with an irrelevant mouse immunoglobulin (MIgG), showed the highest luminescence signal. All E11 antibody treatments significantly reduced luminescence levels (*p* < 0.001), despite a 5-day delay in treatment initiation. The E11-Fab fragment group showed the most profound effect, with the lowest luminescence signal overall. The full E11 mAb and E11-F(ab′)_2_ fragments reduced luminescence by approximately 15-fold and 9-fold, respectively, compared to the control, with no significant difference between them. Flow cytometric analysis, using the DLA-DR specific mAb (B5) for canine tumor cells, confirmed these findings. All E11 antibody variants significantly reduced the CLBL1 tumor cell burden in the bone marrow relative to the MIgG control (Figure 4).

In Table 1, we summarised the experimental outcomes presented above. Data indicate that the full E11-mAb and its fragments significantly increased the proportion of tumor progression-free mice at day 21 (%PR = 50%) compared to the MIgG control group (12%). Time to tumor progression (TTP), assessed based on body weight loss, luminescence increase, and integrated composite indicators, was significantly prolonged in the treated groups relative to controls (*p* < 0.001). The control group exhibited a TTP range of 9.8–12.3 days, whereas mice receiving E11 antibody variants showed extended TTP values around 18–20 days.

During the course of the experiment, a subset of animals—predominantly mice from the group receiving irrelevant mouse IgG antibodies (87.5% of that group, representing 21.8% of all mice included in the study)—met the predefined criteria necessitating humane euthanasia. Mice from the MIgG group reached the clinical condition requiring euthanasia on day 16, whereas four additional animals (*n* = 1 E11-IgG, *n* = 2 E11-F(ab′)_2_, *n* = 1 E11-Fab) met these criteria on day 18 post-tumor cell implantation. Humane endpoints were operationalized as the occurrence of any of the following conditions: body weight loss exceeding 20%, profound debilitation accompanied by progressive apathy, clinical manifestations of severe dehydration (including ocular enophthalmos and reduced skin turgor), or neurological deficits such as paralysis or marked hind-limb paresis, spatial disorientation, and circling behavior. No additional signs consistent with animal distress were detected.

These findings indicate the in vivo intrinsic cytotoxic potency of E11 antibody fragments towards the canine lymphoma cell line CLBL1-sLuc.

## 4. Discussion

Our previous work detailed the generation of two murine monoclonal antibodies directed against dog leukocyte antigen DR (DLA-DR). These antibodies demonstrated both intrinsic cytotoxic properties and the capacity to elicit immune-mediated cell death in canine lymphoma and leukemia cell lines under in vitro conditions and in relevant in vivo models [21,22]. Several other murine and human monoclonal antibodies (mAbs) targeting MHC Class II (MHC II) have also been shown to exert direct cytotoxic effects against B cell neoplasms or activated B cells [19,30]. Significantly, however, no Fab or F(ab′)_2_ fragment is yet FDA-approved for monotherapy in oncology. Nevertheless, smaller fragments are central to several promising cancer strategies, including their use as bispecific T-cell engagers (BiTEs) or components of chimeric antigen receptors (CARs) [31]. For instance, bispecific anti-CD20/CD19 CAR T cells (LV20.19) have demonstrated both low toxicity and high efficacy in treating relapsed/refractory B-cell malignancies (NCT03019055) [32]. Our key finding—the direct killing mechanism by a monovalent E11-Fab fragment—establishes a novel foundation. This intrinsic effector function, coupled with the flexible platform of the Fab fragment, paves the way for a new class of biological reagents that can be further enhanced through conjugation with cytotoxic payloads or T-cell engagers to potentiate anti-tumor effects.

It has been widely accepted that crosslinking of MHC II with full-length antibodies is generally necessary to elicit efficient cell death. Consistent with this, certain bivalent F(ab′)_2_ fragments, although sometimes less potent, have demonstrated cytotoxicity, whereas it has been observed that monovalent Fab fragments do not trigger detectable cytotoxic effects, even when applied at elevated concentrations or maintained in prolonged co-culture conditions [23,30]. Furthermore, as demonstrated by Gross and co-workers, a proper spatial orientation of dimerized MHC II was considered essential for apoptotic signaling, as homotypic aggregation of human leukocyte antigen DR HLA-DRα chains by an antibody L243 induced apoptosis in monocyte cell lines, while heterotypic aggregation of HLA-DRa and DRb chains by staphylococcal enterotoxin A (SEA) did not [33].

In the present report, we provide the first demonstration that monovalent interaction of the E11-Fab fragment with canine CLBL1 and CLB70 cells is capable of initiating direct cytotoxic responses (Figure 1B). The cytotoxic activity of the monovalent E11-Fab fragment is highly unusual. We were able to identify only one other published example of a monovalent interaction with MHC II inducing a direct cell death: T-cell receptor (TCR) engagement with major histocompatibility complex class II (MHC II) expressed on naive B cells by some Th0 cell clones [34].

We hypothesize that the E11-Fab fragment, which recognizes a unique conformational epitope formed by both DLA-DRα and DLA-DRβ chains [21], elicits intracellular signaling events comparable in their downstream effects to those of the aforementioned TCR engagement.

This in vitro observation is strongly corroborated by evidence obtained from an in vivo murine model that confirmed the potent therapeutic activity of the monovalent E11-Fab fragment. We observed significant suppression of both tumor growth and bone marrow metastasis (Figure 3). Intriguingly, the E11-Fab activity was comparable to the full E11 mAb—even though the full antibody (IgG2a) is capable of activating Fc-dependent immune mechanisms—and to the bivalent F(ab′)_2_ fragment, which can still crosslink MHC II molecules. This result strongly suggests that the crosslinking of MHC II by E11 is not critically important for its direct cytotoxic function.

As reported by Vidović et al., the majority of cytotoxic mAbs directed MHC II also possess the capacity to induce the downregulation of DR molecules presented at the plasma membrane of antigen-presenting cells (APCs). This downmodulating capacity is observed for both full antibodies and their monovalent fragments and is generally dependent on the antibody’s binding epitope on MHC II. It has been proposed that cytotoxic anti-MHC II mAbs often target the first, N-terminal, peptide-binding domain of MHC II DR molecules, although not all antibodies binding this region are downmodulating [23].

The binding of E11 mAb to CLBL1 and CLB70 cells is accompanied by a downmodulation of approximately 50% of the initial cell surface expression of MHC II within 4 h of incubation (Figure 2). This level of MHC II downmodulation was significantly lower than that induced by the L243 Fab on EBV-LCL cells (~80%) [23] and by an anti-DLA-DRα Fab B5 on CLBL1 cells (~75%) (Moniakowski, L. and Miazek, A., manuscript in preparation).

These comparative results suggest that the degree of MHC II downmodulation may be directly related to the specific MHC II epitope engaged by the anti-MHC II antibody. For E11 mAb, the conformational epitope requires co-expression of both DLA-DRα and DLA-DRβ chains, whereas the binding sites of L243 and B5 mAbs are within the DRα chain [35,36].

The intermediate level of DR downregulation induced by E11 mAb may be regarded as a favorable feature for potential clinical veterinary use. Since MHC II downmodulation on the surface of APCs inhibits antigen presentation and subsequent T-cell activation, the use of anti-MHC II antibodies to kill B-cell neoplasms carries a risk of adverse effects related to the loss or transient depletion of normal APC function—a kind of “double-edged sword” effect. Published data confirm a transient depletion of peripheral blood HLA-DR-positive APCs following infusion of human anti-MHC II agents like 1D09C and IMMU-114 [37,38]. In this context, the E11-Fab fragment’s lower propensity for downmodulation could better preserve MHC II expression on APCs following infusion and thereby limit systemic immune suppression.

### Study Limitations

While our findings strongly support the intrinsic therapeutic activity of the monovalent Fab fragment targeting DLA-DR, we acknowledge several limitations in the present study that warrant discussion and future investigation.

Our primary in vivo efficacy data were generated using a xenograft mouse model involving immunodeficient NOD-SCID mice transplanted with the canine CLBL1 cell line. This approach, while necessary for controlled head-to-head comparisons of the non-immune-mediated effects of the antibody fragments, inherently presents two limitations: (1) The absence of a functional murine immune system (including B and T cells) prevents us from assessing potential Fc-mediated effector functions (e.g., ADCC or CDC) or evaluating the risk of off-target B cell depletion in vivo. For this reason, we were unable to directly monitor B cell status following treatment, a constraint previously addressed by in vitro data from normal canine PBMCs [21]. (2) Although the MHC II pathway of apoptosis is highly conserved across species, the extrapolation of findings from a canine B-cell line in a murine model to the complexity of human lymphomas requires caution.

While we demonstrate that the Fab fragment is sufficient for apoptosis, our study does not elucidate the precise downstream signaling pathways activated by the single-site DLA-DR engagement. Future work is required to define the role of specific intracellular mediators (e.g., caspases, mitochondrial pathways) that are activated independently of crosslinking.

## 5. Conclusions

In the present study, we provide the first direct evidence that monovalent engagement of the E11-Fab fragment with canine CLBL1 and CLB70 lymphoma cells is sufficient to induce direct cytotoxicity in vitro and can effectively suppress tumor growth in vivo.

Previously, the crosslinking of MHC II molecules by bivalent antibodies or F(ab′)_2_ fragments was generally considered a prerequisite for inducing death signaling in cancerous B cells. Our observation challenges this paradigm and strongly points to the existence of unique conformational epitopes on the DLA-DR heterodimer. The engagement of this specific epitope by a monovalent antibody fragment is sufficient to trigger cell death signaling, functionally analogous to the pathway elicited by TCR-MHC II interactions.

## Figures and Tables

**Table 1:** Comparison of Time to Tumor Progression in CLBL1-sLuc tumor-bearing mice treated with various E11-derived antibody fragments.

Treatment	N	%PR (TF)	TTP Body Weight (Days) ± S.D.	TTP Luminescence (Days) ± S.D.	TTP Combined Signals (Days) ± S.D.	*p* Versus MIgG
MIgG	8	12% (1)	12.3 ± 3.5	9.8 ± 4.7	11.0 ± 4.2	N.A.
E11 -IgG	8	50% (4)	19.1 ± 1.6	18.8 ± 3.3	18.7 ± 2.5	<0.001
E11 -F(ab)2	8	50% (4)	19.3 ± 2.0	18.1 ± 2.9	18.7 ± 2.5	<0.001
E11 -Fab	8	50% (4)	19.3 ± 2.0	19.4 ± 2.6	19.3 ± 2.2	<0.001

N—number of mice in the given group, %PR (TF)—percentage of mice without tumor progression by day 21, defined as animals showing no weight loss or sharp increase in luminescence. TTP (Days) ± S.D.—mean time to tumor progression ± standard deviation. TTP-body weight is defined as the point at which body weight began a sustained decline, with a total loss of at least 10% of the initial weight. TTP-luminescence was determined as the point at which the signal exceeded the 100,000 RLU threshold and subsequently showed an increasing trend in luminescence in peripheral blood. TTP-combined signals: time to progression assessed by integrating both progression indicators-body weight loss and increased blood luminescence; *p*—probability, N.A.—not applicable in the corresponding figures. The *p*-value was calculated using the Mann–Whitney test in GraphPad Prism 8.0.2 with the statistical analysis function for nonparametric tests.

## Data Availability

The datasets from the current study are available from the corresponding author upon reasonable request.

## Supplementary Materials

The following supporting information can be downloaded at: https://www.mdpi.com/article/10.3390/cancers18010048/s1, Figure S1: The uncropped SDS-PAGE. Table S1: Changes in peripheral blood luminescence in mice following cell implantation.

## Abbreviations

The following abbreviations are used in this manuscript:

APCs	Antigen-presenting cells
cDLBL	Canine diffuse large B cell lymphoma
DLA-DR	Dog leukocyte antigen DR
HLA DR	Human Leukocyte antigen DR
MHC II	Major histocompatibility complex class II
SEA	Staphylococcal Enterotoxin A
TTP	Tıme to tumor progression
