# Supplementary material for: Fab Antibody Fragments to Dog Leukocyte Antigen DR (DLA-DR) Directly Suppress Canine Lymphoma Cell Line Growth In Vitro and in Murine Xenotransplant Model
Source: Cancers (Basel). 2025 Dec 23;18(1):48. doi: 10.3390/cancers18010048 (PMC12784876; doi:10.3390/cancers18010048)
Supplement: Supplementary file 1 [file cancers-18-00048-s001.zip › Supplementary Figure S1.pdf]

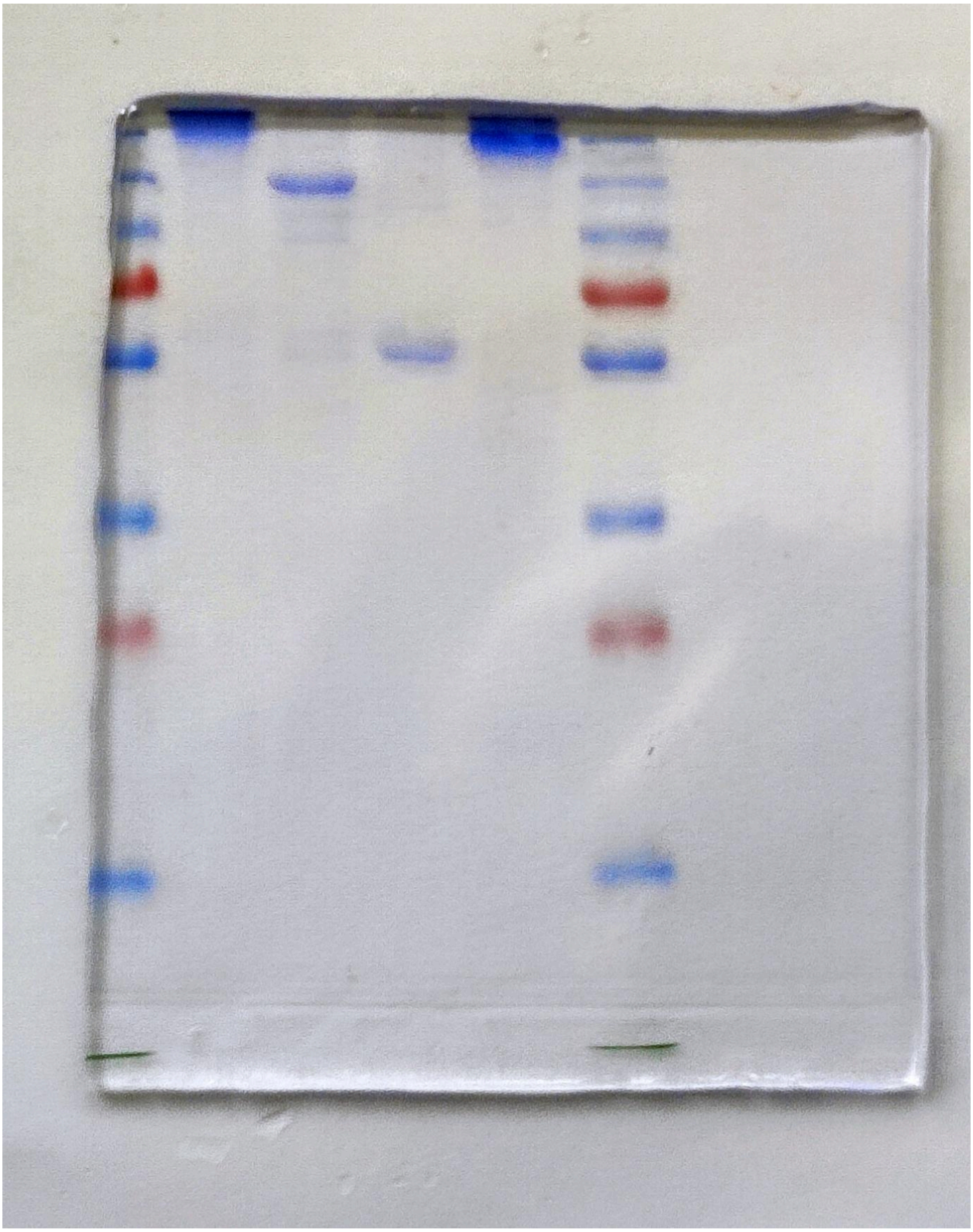

Supplementary Figure S1. Source image of SDS-PAGE that confirms the molecular weights of the full E11 monoclonal antibody (mAb) and its derived fragments.
Original, uncropped SDS-PAGE image used for the preparation of Figure 1(A).
