# Supplementary material for: Fab Antibody Fragments to Dog Leukocyte Antigen DR (DLA-DR) Directly Suppress Canine Lymphoma Cell Line Growth In Vitro and in Murine Xenotransplant Model
Source: Cancers (Basel). 2025 Dec 23;18(1):48. doi: 10.3390/cancers18010048 (PMC12784876; doi:10.3390/cancers18010048)
Supplement: Supplementary file 1 [file cancers-18-00048-s001.zip › Supplementary Table S1.pdf]

**Table S1. Changes in peripheral blood luminescence in mice following cell implantation.**

| **MIgG vs E11-IgG** | | | | |
|---|---|---|---|---|
| **Column Factor** | **Difference** | **t** | **P value** | **Summary** |
| Day 2 | -8784 | 0,04138 | P > 0,05 | ns |
| Day 4 | -4527 | 0,02133 | P > 0,05 | ns |
| Day 7 | -42707 | 0,2012 | P > 0,05 | ns |
| Day 16 | -3,25E+06 | 15,32 | P<0,001 | *** |
| **MIgG vs E11-F(ab')2** | | | | |
| **Column Factor** | **Difference** | **t** | **P value** | **Summary** |
| Day 2 | -13524 | 0,06372 | P > 0,05 | ns |
| Day 4 | -7071 | 0,03331 | P > 0,05 | ns |
| Day 7 | -49794 | 0,2346 | P > 0,05 | ns |
| Day 16 | -3,22E+06 | 15,15 | P<0,001 | *** |
| **MIgG vs E11-Fab** | | | | |
| Column Factor | Difference | t | P value | Summary |
| Day 2 | -294 | 0,001385 | P > 0,05 | ns |
| Day 4 | -9087 | 0,04281 | P > 0,05 | ns |
| Day 7 | -55507 | 0,2615 | P > 0,05 | ns |
| Day 16 | -3,26E+06 | 15,36 | P<0,001 | *** |
| E11-IgG vs E11-F(ab')2 | | | | |
| **Column Factor** | **Difference** | **t** | **P value** | **Summary** |
| Day 2 | -4740 | 0,06147 | P > 0,05 | ns |
| Day 4 | -2544 | 0,03299 | P > 0,05 | ns |
| Day 7 | -7087 | 0,0919 | P > 0,05 | ns |
| Day 16 | 35764 | 0,4638 | P > 0,05 | ns |
| E11-IgG vs E11-Fab | | | | |
| **Column Factor** | **Difference** | **t** | **P value** | **Summary** |
| Day 2 | 8490 | 0,1101 | P > 0,05 | ns |
| Day 4 | -4560 | 0,05913 | P > 0,05 | ns |
| Day 7 | -12800 | 0,166 | P > 0,05 | ns |
| Day 16 | -7206 | 0,09345 | P > 0,05 | ns |
| E11-F(ab')2 vs E11-Fab | | | | |
| **Column Factor** | **Difference** | **t** | **P value** | **Summary** |
| Day 2 | 13230 | 0,1716 | P > 0,05 | ns |
| Day 4 | -2016 | 0,02614 | P > 0,05 | ns |
| Day 7 | -5713 | 0,07408 | P > 0,05 | ns |
| Day 16 | -42970 | 0,5572 | P > 0,05 | ns |

The table presents the statistical significance of differences in peripheral blood bioluminescence measurements between experimental groups at selected time points. Days 2, 4, and 7 correspond to the early phase of the experiment, with time points calculated from the day of inoculation with CLBL1-Luc cells. Day 16 represents the terminal time point for mice in the control group, at which animals were euthanized for humanitarian reasons. Statistical analyses were performed based on peripheral blood bioluminescence measurements, expressed as relative light units (RLU), collected throughout the course of the experiment. Group sizes were as follows: MIgG, $n = 8$; E11-IgG, $n = 8$; E11-F(ab′)$_2$, $n = 8$; and E11-Fab, $n = 8$. Statistical significance was assessed using two-way analysis of variance (two-way ANOVA) conducted with GraphPad Prism software version 8.0.2.

Based on the obtained results, we demonstrate that the experimental groups were homogeneous following cell implantation. Differences between the control group and the treated groups emerged progressively over the course of the experiment. No statistically significant differences were observed up to day 7 between the control group (MIgG) and the groups receiving E11 antibody–based therapy (E11-IgG, E11-F(ab′)$_2$, and E11-Fab).

Importantly, on day 16, luminescence levels - used as an indicator of disease progression - differed significantly between the control group and all treated groups ($p < 0.001$). In contrast, no statistically significant differences were detected among the E11-treated groups themselves, indicating comparable therapeutic efficacy of the tested E11 antibody formats.
